# Combined use of 18 F-FDG PET/CT and MRI for evaluating infection status and clinical outcomes before reimplantation in two-stage revision arthroplasty

**DOI:** 10.1186/s13018-026-06713-7

**Published:** 2026-02-16

**Authors:** Gürhan Tükel, Vasfi Karatosun, Recep Bekiş, Nazlı Pınar Karahan Şen

**Affiliations:** 1 Department of Orthopaedics and Traumatology, Milas State Hospital, 48200 Muğla, Turkey; 2 Department of Orthopaedics and Traumatology, Dokuz Eylul University Faculty of Medicine, 35330 İzmir, Turkey; 3 Department of Nuclear Medicine, Dokuz Eylul University Faculty of Medicine, 35330 İzmir, Turkey

**Keywords:** Periprosthetic joint infection, Arthroplasty, Replacement, Revision, Positron-Emission tomography computed tomography, Magnetic resonance imaging, Infection control

## Abstract

**Background:**

Two stage revision arthroplasty (TSRA) is the standard treatment for periprosthetic joint infections (PJI); however, the utility of imaging in determining infection status before reimplantation remains unclear. The aim of this study was to evaluate whether pre-reimplantation 18 F-Fluorodeoxyglucose positron emission tomography/computed tomography (18F-FDG PET/CT) and magnetic resonance imaging (MRI) findings are associated with the risk of recurrent infection after second-stage revision arthroplasty.

**Methods:**

A total of 47 patients (26 women and 21 men) diagnosed with PJI according to the 2011 Musculoskeletal Infection Society (MSIS) criteria and treated with TSRA were retrospectively included in the study. A total of 26 hip and 21 knee PJI cases were evaluated. 18F-FDG PET/CT and MRI scans were obtained before reimplantation, and the images were interpreted by a radiologist and a nuclear medicine specialist blinded to the patient’s clinical outcomes. 18F-FDG PET/CT and MRI findings were compared with postoperative infection outcomes.

**Results:**

After reimplantation, no infection was observed in 33 patients, whereas 14 patients experienced infection recurrence requiring surgical intervention. 18F-FDG PET/CT alone identified 31 patients as infected and 16 as non-infected. 18F-FDG PET/CT results significantly correlated with clinical outcomes (*p* = 0.017). MRI alone identified 20 patients as infected and 27 as non-infected. A statistically significant association was observed between MRI findings and clinical outcomes (*p* = 0.009). When 18F-FDG PET/CT and MRI findings were combined, diagnostic accuracy improved, yielding a sensitivity of 100% and negative predictive value (NPV) of 100%.

**Conclusion:**

A negative 18F-FDG PET/CT and MRI scan can provide an important reference point, strongly suggesting infection eradication and supporting a decision to proceed with the second stage of reimplantation surgery.

## Introduction

 Periprosthetic joint infection (PJI) remains one of the most challenging complications following total joint arthroplasty, given its indolent presentation and the difficulties associated with accurate diagnosis and effective management. In-hospital mortality due to PJI–related systemic inflammatory response syndrome or septic shock has been reported to be approximately 3.5% in large revision cohorts, with a rapidly increasing risk in severe cases, highlighting once again the critical importance of timely diagnosis and appropriate treatment [1]. Two-stage revision arthroplasty (TSRA) has long been established as a standard procedure for eradicating infection while preserving joint function [2–4]. The procedure involves prosthesis removal, thorough debridement, and placement of an antibiotic loaded spacer during the first stage, followed by reimplantation after completion of antibiotic therapy and presumed infection eradication [5]. Patients with PJI represent a challenging group for reimplantation arthroplasty due to reduced bone stock, wound complications resulting from multiple previous surgeries, and prolonged treatment courses. Therefore, particular caution is required, as these patients are at an increased risk for recurrent infection following reimplantation [6]. However, timely determining the infection status before reimplantation remains a significant challenge in clinical practice. To date, no definitive biomarker or diagnostic test has been identified in the literature which can reliably guide the timing or decision for reimplantation [7–9].

Functional imaging with 18 F-fluorodeoxyglucose positron emission tomography/computed tomography (18F-FDG PET/CT) has demonstrated high sensitivity and moderate specificity for detecting PJI. However, its role in evaluating infection status before reimplantation is less clearly defined [10, 11]. Only a few studies have evaluated using 18F-FDG PET/CT for assessing infection status before reimplantation arthroplasty (see Chen et al. and Huang et al.) [12].

Recently, newly developed magnetic resonance imaging (MRI) techniques have introduced metal artifact reduction sequences (MARS**)**, enabling reliable evaluation of postoperative complications (e.g., infection, soft tissue inflammation, or pseudotumor formation) following arthroplasty [13, 14]. These advancements suggest that MRI may be a promising imaging modality for evaluating infection status before reimplantation in patients undergoing TSRA, though the use of this morphologic technique has not yet been thoroughly investigated. This study aimed to evaluate the diagnostic performance of 18F-FDG PET/CT and MRI in determining infection status before reimplantation in patients undergoing TSRA and to evaluate the clinical impact of combining these imaging modalities.

## Patients and methods

### Study design and patient selection

This retrospective study included patients who underwent TSRA for PJI between 2011 and 2023, all performed by a single surgeon at the author’s university hospital’s department of Orthopedics and Traumatology. Patients who underwent 18F-FDG PET/CT and contrast-enhanced MRI to assess infection status before reimplantation were included. ‘Exclusion criteria’ were culture positivity by aspiration before reimplantation, history of malignancy, previous PJI in another joint, presence of intraoperative purulence at the reimplantation stage, loss to follow-up after both surgical stages, renal dysfunction preventing contrast imaging, or missing clinical or radiological data.

Demographic characteristics, comorbidities, laboratory results, imaging findings, and operative and follow-up records were retrospectively reviewed. 47 patients met the inclusion criteria 26 with hip and 21 with knee arthroplasties. Only one joint per patient was analyzed. This study was approved by the Institutional Review Board of Dokuz Eylül University Faculty of Medicine (IRB No: 2023/31-9). The requirement for informed consent was waived due to the retrospective nature of the study.

### Diagnosis of PJI and first stage procedure

The diagnosis of PJI was established according to the 2011 MSIS criteria [15]. Among the cohort, 12 patients were diagnosed based on a *sinus tract* communicating with the prosthesis, five based on *two positive cultures* with the same organism, and 30 by fulfilling the 2011 *MSIS minor criteria*. At least five tissue samples were obtained from separate periprosthetic sites for microbiological culture and histopathological analysis during the first stage procedure. The prosthesis was removed after sample collection, and extensive debridement with pulsatile lavage was performed. Antibiotic loaded spacers were inserted in all cases. Patients with culture-positive results received spacers containing pathogen specific antibiotics, whereas vancomycin (2 g per 40 g bone cement) was administered in culture-negative cases.

### Antibiotic therapy and pre-reimplantation imaging

Following the first stage procedure, all patients received a standardized ‘six-week course’ of intravenous antibiotics prescribed by an infectious disease and clinical microbiology specialist. Oral antibiotic therapy was continued when indicated based on clinical and laboratory findings. After completion of antimicrobial treatment, an antibiotic free interval—a holiday period–of at least two weeks was observed before reimplantation. Patients were monitored with serial measurements of CRP, ESR and procalcitonin during the whole treatment period. Finally, the decision for reimplantation was solely based on the patients’ clinical findings and serum inflammatory marker levels, thus without use of the combined imaging results.

Preoperative aspiration fluid were obtained from all patients prior to reimplantation.

In some patients, sufficient synovial fluid could not be obtained for analysis, therefore only culture was performed in all patients. Microbiological cultures were considered positive when a clinically relevant, highly virulent organism was isolated, or when growth of common skin commensals (e.g., coagulase-negative staphylococci) was supported by concordant clinical and laboratory findings. Single colony-forming unit growth of common skin commensals without supportive clinical or laboratory evidence was regarded as contamination. Patients without clinical signs of local infection, a serum CRP level below 15 mg/L, and negative aspiration cultures were deemed eligible for reimplantation by an infectious disease and clinical microbiology specialist. A CRP cut-off of 15 mg/L was adopted to include patients with subclinical infections and to account for elevated CRP values potentially caused by comorbidities. Patients with positive aspiration cultures prior to reimplantation were not subjected to the second-stage procedure and were excluded from the study.

**Fig. 1:**
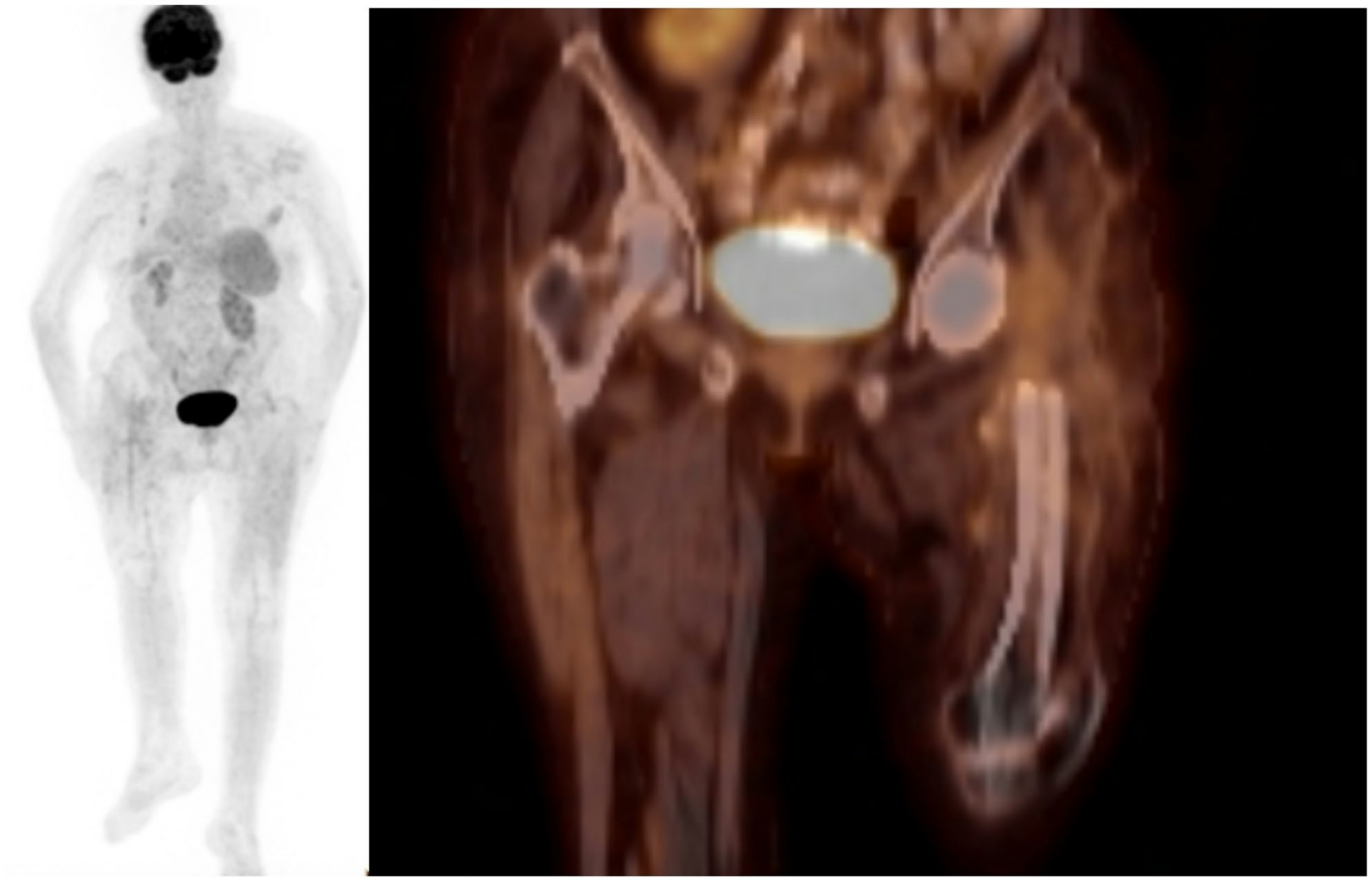
(Negative example) Representative 18F-FDG PET/CT images demonstrating diffuse, homogeneous periarticular FDG uptake without focal activity at the bone–cement interface, classified as non-suggestive of infection according to the predefined criteria. (SUV-max: 2.7)

**Fig. 2:**
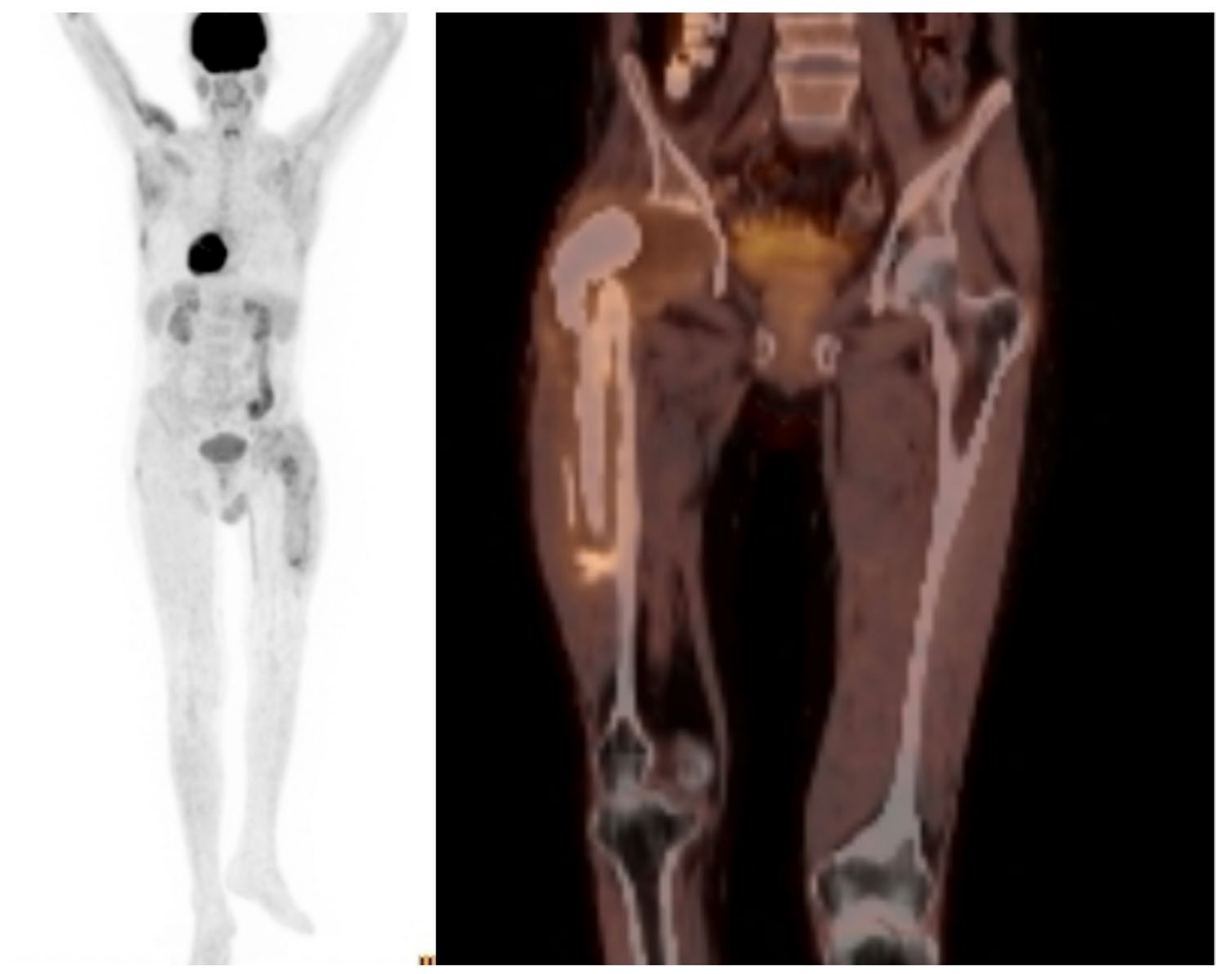
(Positive example). Axial and coronal fused 18F-FDG PET/CT image demonstrating focal increased FDG uptake at the bone–cement interface, considered suggestive of infection according to the predefined PET criteria. (SUV-max: 3.5)

Prior to the second-stage procedure, 18F-FDG PET/CT was performed in all patients to evaluate imaging findings that could indicate residual disease and be associated with an increased risk of reinfection following reimplantation. The imaging studies were reviewed by a nuclear medicine specialist who was blinded to the clinical outcomes, using diagnostic criteria reported in the literature [16–18]. FDG uptake at the bone–cement interface, particularly when the uptake intensity exceeded that of the surrounding background tissue, was considered suggestive of infection. In contrast, diffuse and homogeneous FDG uptake confined to periarticular soft tissues or consistent with postoperative inflammatory changes was not considered suggestive of infection. The infection status (positive or negative) and the maximum standardized uptake value (SUV-max) were recorded for each patient (Figs. 1 and 2).

Within one week after 18F-FDG PET/CT, the affected joint’s contrast-enhanced MRI was performed using a ‘1.5-T scanner’. The imaging protocol included T1-weighted, T2-weighted, and proton density weighted (PDW) sequences encompassing the entire joint. MRI findings suggestive for infection included synovitis, extracapsular fluid collection, and abscess formation [13, 19]. All MRI images were interpreted by a radiologist blinded to clinical outcomes, and infection status (positive or negative) around the spacer was documented accordingly.

### Post-reimplantation period and follow-up

Tissue cultures were taken from at least three different anatomic sites at the time of reimplantation, whereas intraoperative frozen section analysis was not performed. Patients in whom purulence was observed during the reimplantation procedure did not undergo reimplantation and were excluded from the study. To prevent ‘surgical site infection’ following reimplantation, prophylactic cefazolin was administered at a dosage of 1 g twice daily for 2 days. During the first three months following reimplantation, patients with persistent wound drainage (> 7 days), a sinus tract communicating with the prosthesis, or positive joint aspirate cultures were classified as clinically infected and were prone for surgical debridement. Patients with no evidence of infection during follow-up (nice wound healing, no discharge, no fistula) were classified as clinically uninfected. The follow-up period was the time between second-stage reimplantation and the last clinical evaluation.

### Statistical analysis

All statistical analyses were performed using IBM SPSS^®^ Statistics software, version 27.0 (IBM Corp., Armonk, NY, USA). Categorical variables were summarized as frequencies and percentages, whereas continuous variables were presented as means with standard deviations or medians with interquartile ranges, as appropriate. Group comparisons were conducted using Fisher’s exact or Pearson’s chi-square test for categorical data and the Wilcoxon rank-sum test for continuous variables. A two-tailed *p*-value < 0.05 was considered statistically significant.

## Results

The study included 47 patients who underwent TSRA, comprising 26 hips (55.3%) and 21 knees (44.7%). The mean age of the patients was 65.5 ± 12.8 years (range, 35–86 years), with 26 females (55.3%) and 21 males (44.7%).

‘Comorbidities’ were present in most patients, with hypertension being the most common (62.5%). Seventeen patients who did not show sufficient resolution of infection after the first-stage surgery underwent an additional debridement procedure. The mean interval between the first-stage procedure and 18F-FDG PET/CT was 103.3 ± 35.1 days (range, 49–260 days), and the mean interval between imaging and second-stage reimplantation was 52.6 ± 43.1 days (range, 6–181 days). After reimplantation, the mean follow-up duration was 44.9 ± 35.3 months (range, 5–120 months). The mean time to reinfection after reimplantation was 21.7 ± 9.9 days (range, 12–42 days).

Among patients without reinfection after reimplantation (*n* = 33), the mean pre-reimplantation CRP level was 10.8 ± 4.5 mg/L (range, 0.9–13.2 mg/L). In patients who developed reinfection (*n* = 14), the mean CRP level was 13.2 ± 5.5 mg/L (range, 0.9–15.0 mg/L). Although CRP values were higher in the reinfection group, the difference was not statistically significant (*p* = 0.17).

Based on the tissue cultures obtained during the first-stage surgery, microbial growth was detected in 23 patients (48.9%), while no growth was observed in 24 patients (51.1%). The most frequently isolated pathogen was coagulase-negative Staphylococcus spp., identified in five patients. At the time of reimplantation, tissue cultures revealed microbial growth in 3 patients (6.3%), while no growth was detected in 44 patients (93.7%). In all patients with microbial growth in cultures obtained during the reimplantation procedure, the isolated organisms were different from those identified during the first-stage surgery, and all three of these patients developed infection during follow-up. (Table 1)

Among the 14 patients who developed infection after reimplantation, comparison of first-stage and post-reimplantation debridement cultures revealed the same microorganism in 5 patients, a different microorganism in 4 patients, while cultures were negative at both stages in the remaining 5 patients. (Table 2)

**Table 1:** At the first stage, cultures were negative in 25 patients (53.2%) at reimplantation, cultures were negative in 44 patients (93.6%), with only three patients showing positive Microbiological findings

First-stage culture microorganism	
No growth	25 (53.2%)
CoNS spp.	6 (12.8%)
A. baumannii	4 (8.5%)
P. aeruginosa	2 (4.3%)
S. aureus	3 (6.4%)
E. faecalis	2 (4.3%)
M. tuberculosis	1 (2.1%)
C. striatum	1 (2.1%)
S. epidermidis	1 (2.1%)
Enterobacter spp.	1 (2.1%)
S. pyogenes	1 (2.1%)

Second-stage culture microorganism	
No growth	44 (93.6%)
CoNS spp.	2 (4.3%)
S. epidermidis	1 (2.1%)

CoNS = coagulase-negative staphylococc

Percentages are calculated based on the total number of patients

**Table 2:** Comparison of first stage and post-reimplantation debridement culture results in patients with post-reimplantation infection

Patient No	Age / Sex	First-stage culture	Debridement culture	Interpretation
6	50 / M	Mycobacterium tuberculosis	Mycobacterium tuberculosis	Relapse
8	72 / F	Pseudomonas aeruginosa	Pseudomonas aeruginosa	Relapse
10	54 / M	Acinetobacter baumannii	CoNS spp.	Reinfection
12	47 / M	No growth	No growth	Indeterminate
13	54 / M	No growth	No growth	Indeterminate
18	47 / M	No growth	No growth	Indeterminate
24	79 / M	CoNS spp.	CoNS spp.	Relapse
29	67 / F	Staphylococcus epidermidis	CoNS spp.	Reinfection
30	79 / F	No growth	No growth	Indeterminate
32	86 / F	Acinetobacter baumannii	Acinetobacter baumannii	Relapse
33	63 / M	CoNS spp.	Staphylococcus epidermidis	Reinfection
41	78 / F	No growth	No growth	Indeterminate
42	40 / M	CoNS spp.	CoNS spp.	Relapse
46	78 / F	No growth	Staphylococcus aureus	Reinfection

Relapse was defined as recurrence caused by the same microorganism isolated at the first stage

Reinfection was defined as infection caused by a different microorganism

Cases with negative cultures at both stages were classified as indeterminate

CoNS = coagulase-negative staphylococc

Based on 18F-FDG PET/CT findings, 31 patients (66.0%) were classified as ‘infection-positive’, and 16 patients (34.0%) as ‘infection-negative’. Among those with negative scans, 93.8% remained clinically infection-free, whereas patients with positive scans only 41.9% where eventually proven for infection. A statistically significant association was observed between 18F-FDG PET/CT results and clinical outcomes (*p* = 0.017). The sensitivity, specificity, positive predictive values (PPV) and negative predictive values (NPV) of 18 F- FDG PET/CT for detecting spacer infection were 92.8, 45.4, 42.0, and 94.0%, respectively (Table 3).

**Table 3:** Relationship between 18F-FDG PET/CT findings and clinical outcomes

18F-FDG PET/CT result	Clinically infected (n, %)	Clinically not infected (n, %)	Total (n, %)	*p*
Infection compatible	13 (41.9%)	18 (58.1%)	31 (66%)	
Infection incompatible	1 (6.2%)	15 (93.8%)	16 (34%)	
Total	14 (29.8%)	33 (70.2%)	47 (100%)	0.017

**Fig. 3:**
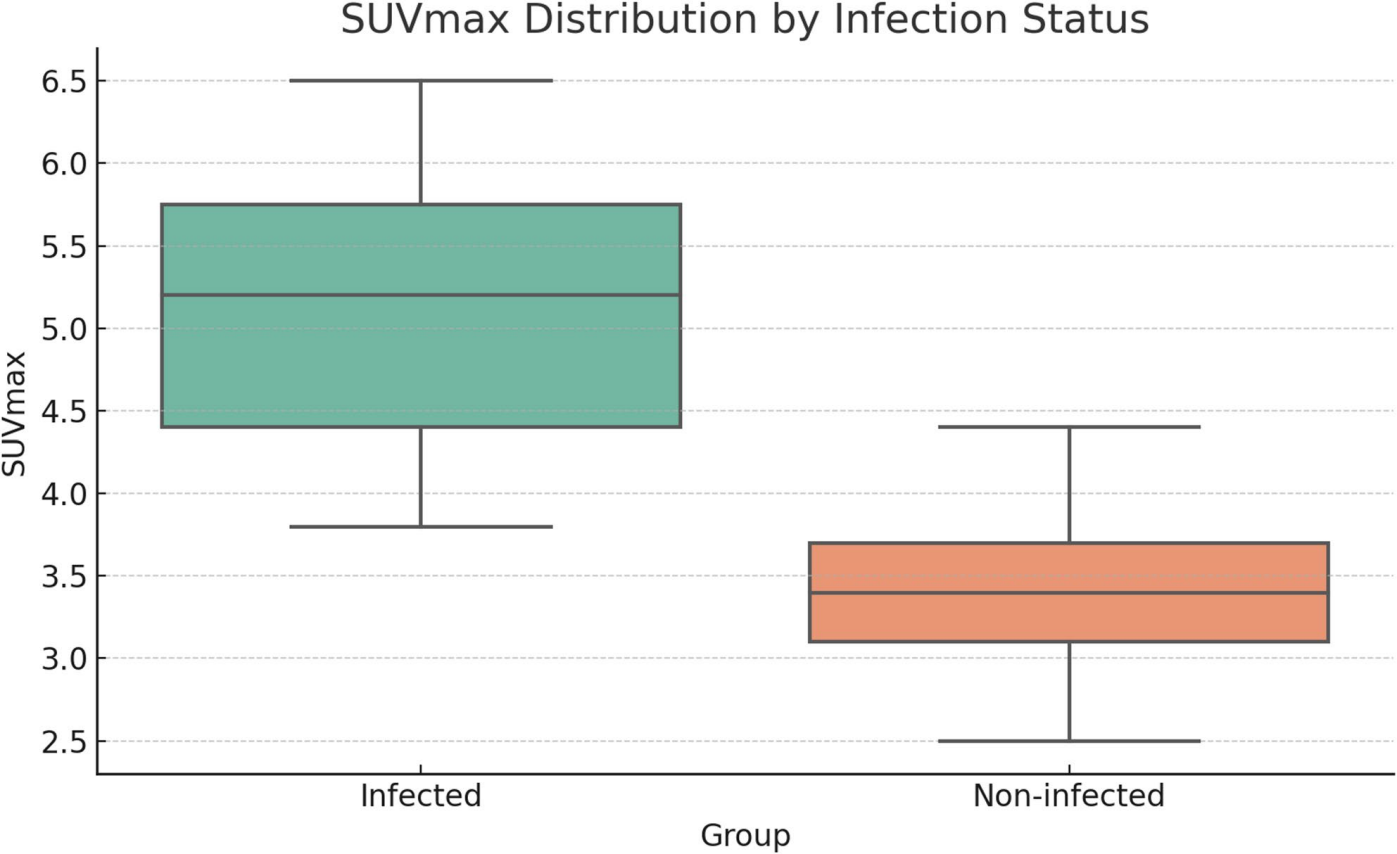
Distribution of SUVmax by infection status. The infected group showed a higher mean SUVmax, but the difference was not statistically significant (*p* = 0.14)

Patients who developed reinfection after reimplantation demonstrated higher mean SUV-max values (5.10 ± 1.95) than those without reinfection (3.50 ± 1.80) (Fig. 3). However, this difference did not reach statistical significance (*p* = 0.14).

Based on MRI findings, 20 patients (42.5%) were classified as ‘infection-positive’ and 27 (57.5%) as ‘infection-negative’. Among those with negative MRI results, 85% remained clinically infection-free, whereas patients with positive MRI results only 50% where eventually proven for infection. MRI findings demonstrated a statistically significant correlation with clinical outcomes (*p* = 0.009). MRI’s sensitivity, specificity, PPV and NPV for detecting spacer infection were 71.0, 69.0, 50.0, and 85.0%, respectively (Table 4).

**Table 4:** Relationship between MRI results and clinical outcomes

MRI result	Clinically infected (n, %)	Clinically not infected (n, %)	Total (n, %)	*p*
Infection Compatible	10 (50%)	10 (50%)	20 (42.5%)	
Infection Incompatible	4 (14.9%)	23 (85.1%)	27 (57.5%)	
Total	14 (29.8%)	33 (70.2%)	47 (100%)	0.009

**Fig. 4:**
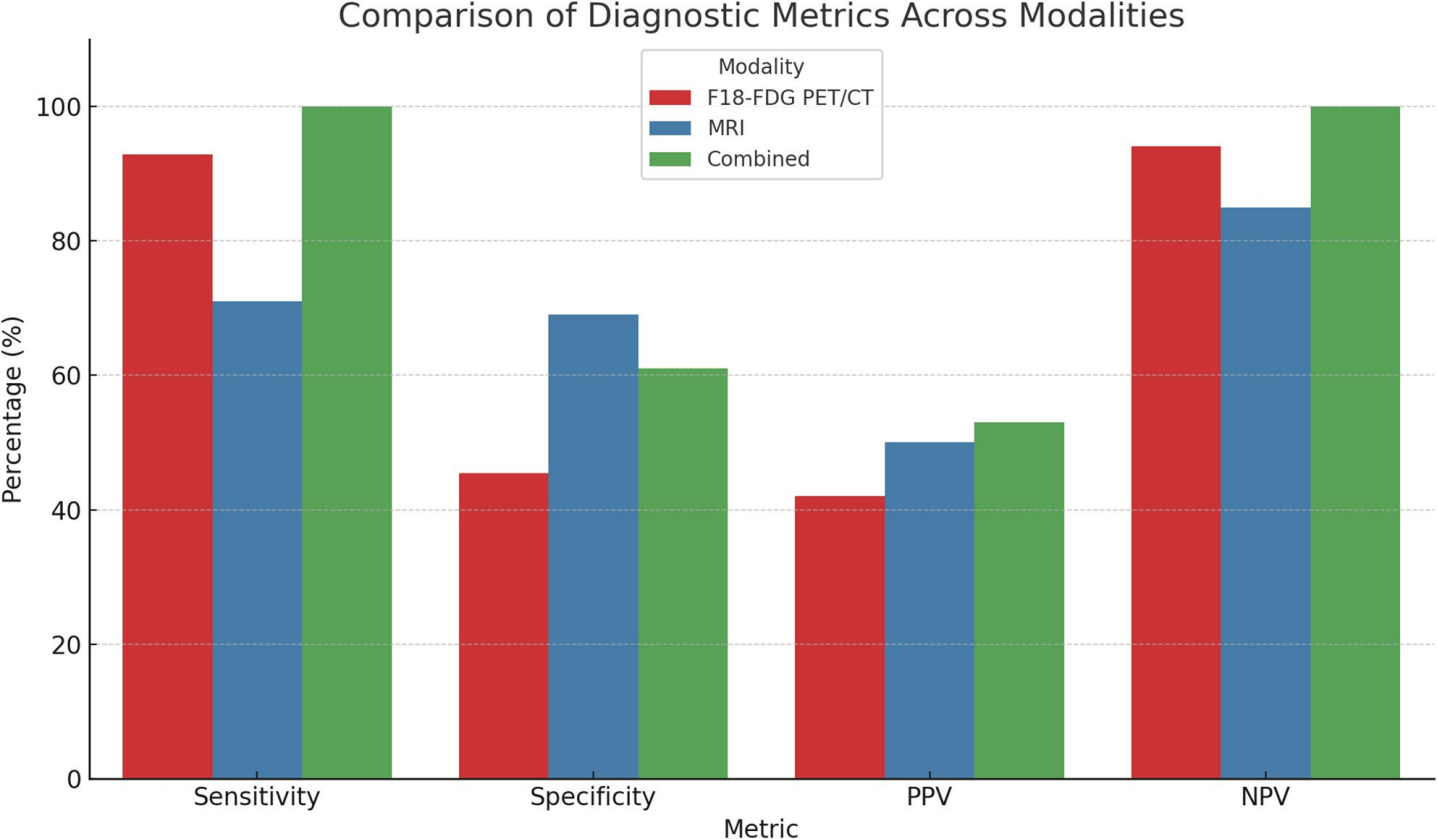
Combined 18F-FDG PET/CT and MRI demonstrated the highest sensitivity and NPV, indicating substantial diagnostic value when both modalities agree

When 18F-FDG PET/CT and MRI results were evaluated, 15 patients (31.9%) showed ‘infection-positive’ findings, and 11 (23.4%) showed ‘infection-negative’ findings in both modalities. Among patients with concordant imaging results, combined interpretation of 18F-FDG PET/CT and MRI demonstrated a statistically significant association with clinical outcomes for infection detection (*p* = *0.002*) and exclusion (*p* = *0.014*). The combined imaging approach yielded a sensitivity of 100.0%, specificity of 61.0%, PPV of 53.0%, and NPV of 100.0% (Table 5; Fig. 4).

**Table 5:** Relationship between results of 18F-FDG PET/CT and MRI

Test	PET/CT	MRI	PET/CT + MRI
Sensitivity	92.8%	71%	100%
Specificity	45.4%	69%	61%
NPV	94%	85%	100%
PPV	42%	50%	53%
Accuracy	59.6%	70.2%	76.9%

NPV: Negative predictive value; PPV: Positive predictive value; PET/CT: Positron emission tomography/Computed tomography; MRI: Magnetic resonance imaging

## Discussion

The reimplantation stage in TSRA represents a critical decision point, as reactivation of infection can lead to new complications and compromise the outcome. In clinical practice, the decision for reimplantation is usually guided by the resolution of clinical symptoms such as diminishing pain, healing of the wound, and the absence of infection signs as well as normalization of serum inflammatory markers. However, these laboratory markers can be influenced by confounding factors, including comorbidities and systemic inflammatory conditions (false positive) or in the case of low-grade infections (false negative) [8, 20]. Consistent with previous studies demonstrating the limited predictive utility of CRP alone for determining reimplantation timing [16] our results showed no statistically significant difference in CRP levels between patients with and without reinfection. These findings underscore the need to integrate clinical, laboratory, and imaging data rather than relying on a single parameter for clinical decision-making process.

Advanced imaging techniques have been largely investigated for the diagnosis of PJI; however, their use for evaluating infection status prior to reimplantation in TSRA has been explored in only a limited number of studies.

*Chen et al.* reported, in a preliminary study of using PET/CT in hip prosthesis infections, a high sensitivity but a lower specificity, in predicting residual infection after insertion of an interim spacer [21]. The authors suggested that the high negative predictive value makes 18F-FDG PET/CT useful for patients who have persistently elevated C-reactive protein (CRP) levels but no other clear signs of infection [21].

*Huang et al.* reported also high sensitivity and NPV in excluding persistent infection in a small cohort of hip PJIs evaluated before reimplantation. Of the 11 staged reimplantation THAs, only 1 reinfection was noted at an average follow-up of 48 months. The success rate of 91% suggests 18F-FDG PET/CT could help in the differential diagnosis of infection around cement spacers, especially in patients with normal clinical findings but elevated CRP levels [12]. Our study expands upon these findings by including a larger and more heterogeneous cohort involving both hip and knee infections, and by incorporating MRI correlation, which enhances confidence in interpretation. But in contrary to the 2 aforementioned small reports, in our study the patients with elevated CRP level were excluded from analysis and therefore were labeled as ‘latent infection’. Our study confirmed high sensitivity and NPV, reaching up to 100% when MRI was used in combination with 18F-FDG PET/CT; however, specificity remained relatively low. Nevertheless, the high NPV indicates that a negative 18F-FDG PET/CT finding is highly effective in assessing the risk of post-reimplantation infection, thereby allowing clinicians to use these imaging modalities as risk stratification tools and potentially avoid unnecessary interventions. A comparative overview of studies assessing residual infection with 18F-FDG PET/CT before reimplantation, including Chen et al., Huang et al., and our current study, is provided in Table 6.

The relatively low specificity of 18F-FDG PET/CT observed in our study may be explained by postoperative inflammatory changes, reparative granulation tissue, and foreign-body reactions associated with antibiotic-loaded spacers, all of which may lead to increased FDG uptake despite the absence of active infection. Similar phenomena have been reported in previous studies evaluating 18F-FDG PET/CT after spacer implantation [21]. Differences between hip and knee prostheses may also contribute to variability in specificity. Knee arthroplasty is generally associated with greater soft-tissue disruption, larger spacer surfaces, and higher mechanical stress related to the spacer compared with hip arthroplasty, which may result in more pronounced nonspecific inflammatory FDG uptake. These factors may partially explain the discrepancy between our findings and those reported by Huang et al., who demonstrated higher specificity in a more homogeneous and smaller cohort.

Little is known about the role of 18F-FDG PET/CT for assessing the treatment response in inflammatory or infectious diseases, especially in osteomyelitis. The important issue that needs to be worked out is differentiation between post-surgical inflammation (normal healing process) and persistent infection. *Jones-Jackson et al.* used a rabbit osteomyelitis model and a dual-time point 18F-FDG PET/CT protocol to differentiate between postsurgical inflammation and infection of the bone. Comparisons were made between non-infected and infected rabbits in which infection with Staph. aureus was induced at the time of surgery. Increased FDG uptake was evident in the bone of all rabbits on day 1 after surgery. The intensity of the FDG uptake, however, could not be used to distinguish between the infected and uninfected groups until day 15 [22]. Laboratory data in rabbits suggest that uncomplicated bone healing may demonstrate increased FDG uptake for up to 6 weeks after injury before returning to normal, whereas osteomyelitis is likely to demonstrate continuously rising SUVs [23].

**Table 6:** Comparison of studies evaluating residual infection with FDG-PET/CT before reimplantation

Study	Country	Year	Sample size	Subject	SN (%)	SP (%)	Acc (%)	Comments
Chen et al.	Taiwan	2010	24 hips	Residual infection before reimplantation with interim spacer	100	63	NR	The high negative predictive value of PET/CT scans is useful to rule out infections in patients with persistently elevated CRP levels.
Huang et al.	Taiwan	2011	13 hips	Detection of infection around antibiotic-loaded cement spacers in patients with elevated CRP	100	100	100	A negative FDG-PET study could be used as an important reference for second- stage reimplantation, whereas a positive FDG-PET study should encourage further treatment to eradicate infection before re- implantation.
Current study	Türkiye	2025	47 (hip & knee)	Risk assessment for residual/latent infection before reimplantation	92.8	45.4	59.6	PET/CT and MRI before reimplantation may assist in guiding reimplantation decisions by identifying patients at risk of residual infection.

SN: Sensitivity; SP: Specificity; Acc: Accuracy; NR: Not reported

In clinical practice, during the initial phase of wound healing, the ‘FDG-avid’ formed granulation tissue gradually decreases through apoptosis and will eventually be replaced by ‘non-FDG avid’ fibrotic scar tissue approximately two months after traumatic injury [24]. That’s the reason why increased FDG uptake during normal wound healing tends to gradually decline over time as inflammation subsides [25]. Therefore, it is recommended that PET/CT imaging be performed at least ‘6–8 weeks’ after surgery, when acute post-surgical inflammation has subsided [26, 27].

Although 18F-FDG PET/CT enables functional assessment through the SUV-max, no universally accepted cutoff for diagnosing infection has been established [16]. Prior studies reported that SUV-max alone has limited ability to distinguish infection from aseptic loosening [28]. Another study has reported that, in the diagnosis of hip and knee prosthetic joint infections, the location of 18 F-FDG uptake is more critical than the intensity of uptake [18]. In our study, though ‘infection-positive’ patients has a higher SUV-max value compared to the ‘infection-negative’ ones, this difference was not statistically significant. This trend suggests that SUV-max may serve as a supportive, semi‑quantitative marker of inflammatory uptake rather than a ‘definitive’ diagnostic criterion assisting further clinicians in evaluating the severity of infection.

Magnetic resonance imaging (MRI) has been increasingly applied in evaluating PJI; however, published evidence regarding its diagnostic performance and clinical application remains limited [29, 30]. Introducing new metal artifact reduction sequences (MARS) has markedly improved image quality by minimizing metallic distortion, enhancing diagnostic confidence. With continued technical refinement, MRI is expected to play an ‘expanding’ role in assessing PJI [31, 32]. To date, no studies in the literature have evaluated the use of MRI alone or in combination with 18F-FDG PET/CT to assess infection status before reimplantation in TSRA. When 18F-FDG PET/CT and MRI findings were combined, the diagnostic performance improved substantially, achieving a sensitivity and NPV of 100% and a moderate specificity of 61%. These findings suggest that an integrated imaging approach may enhance pre‑reimplantation evaluation, particularly in complex or equivocal cases.

Integrated 18 F-FDG PET/MRI, which combines simultaneous metabolic imaging with high-resolution MRI, enables detection of infection-related changes and provides detailed morphological assessment of the extent of PJI, including osseous and soft-tissue involvement. In a preliminary study of 13 patients, Henkelmann et al. reported 100% sensitivity and 100% specificity for simultaneous 18 F-FDG PET/MRI in the diagnosis of PJI [33]. To date, the use of 18 F-FDG PET/MRI prior to reimplantation arthroplasty has not been specifically investigated in the literature. Nevertheless, integrated 18 F-FDG PET/MRI may emerge as a promising imaging modality by simultaneously providing metabolic and high-resolution anatomical information, potentially reducing the need for separate PET and MRI examinations in selected patients.

Although the use of 18F-FDG PET/CT and MRI has increased substantially due to recent advances in imaging technology and software [34, 35], their cost and limited availability continue to restrict routine clinical use [36]. Studies have shown that the management of PJI is associated with increased surgical burden, prolonged hospital stay, and higher healthcare costs [37]. Patients undergoing TSRA represent a particularly vulnerable group with limited opportunities for successful reimplantation due to compromised bone stock and soft-tissue conditions resulting from multiple prior surgeries; therefore, careful consideration of the risk of recurrent infection after reimplantation is essential. The use of 18F-FDG PET/CT and MRI within the two-stage treatment protocol may contribute to reduced relapse rates, fewer reoperations, and fewer subsequent complications, suggesting potential long-term cost-effectiveness.

This study has several limitations that should be acknowledged, including its retrospective nature. Although statistically significant results were obtained, the heterogeneity of the patient population and the absence of randomization may limit generalizability. Persistent postoperative 18 F‑FDG uptake secondary to inflammatory processes such as aseptic inflammatory processes could confound image interpretation, and the lack of a standardized interval between the first‑stage procedure and 18F-FDG PET/CT acquisition may have introduced variability. Moreover there is no universally accepted protocol and interpretation criteria for analysing 18F-FDG PET/CT images. Despite these shortcomings, 18F-FDG PET/CT use is increasing for persistent prosthetic pain without radiographic loosening or reliable infection indicators as defined by Musculoskeletal Infection Society (MSIS) criteria. Furthermore, Kirschner wires or other metallic components within spacers may have generated artifacts in MRI and 18F-FDG PET/CT images, potentially affecting diagnostic accuracy. In this study, metallic spacer implants were not used as an exclusion criterion, and MARS sequences were not applied, which may represent additional confounders. Another limitation of this study is that metal artifact reduction sequence (MARS) technology is not routinely available early in patient recruitment; Therefore, MARS MRI could not be applied consistently to the entire cohort. There is a possibility that the diagnostic performance of the tests will increase with future studies using MARS MRI.

It should also be acknowledged that advanced imaging methods generally show higher diagnostic performance in clinically apparent infections compared with clinically occult infections; This may partially explain the low specificity observed in equivocal cases.

## Conclusion

18F-FDG PET/CT combined with MRI are highly sensitive to detect latent infections in interim prosthetic hip and knee spacers. The high negative predictive value is useful to rule out infections also in patients with low CRP levels. It seems to be that both advanced imaging modalities might serve as an auxiliary tool to exclude latent infections in patients posing a clinical diagnostic dilemma.

## Data Availability

The datasets used and/or analysed during the current study are available from the corresponding author on reasonable request.

## Abbreviations

TSRA: Two stage revision arthroplasty
PJI: Periprosthetic joint infections
18F-FDG PET/CT: 18 F-fluorodeoxyglucose positron emission tomography/computed tomography
MRI: Magnetic resonance imaging
MSIS: Musculoskeletal infection society
MARS: Metal artifact reduction sequences
PPV: Positive predictive values
NPV: Negative predictive values
SUV-max: Standardized uptake value

## References

[1] Migliorini F, Weber CD, Bell A, et al. Bacterial pathogens and in-hospital mortality in revision surgery for periprosthetic joint infection of the hip and knee: analysis of 346 patients. Eur J Med Res. 2023;28(1):177. 37208700 10.1186/s40001-023-01138-y PMC10197383

[2] Franceschini M, Pedretti L, Cerbone V, Sandiford NA. Two stage revision: indications, techniques and results. Ann Joint. 2022;7:4. 10.21037/aoj-20-84 PMC10929304 38529148

[3] Insall JN, Thompson FM, Brause BD. Two-stage reimplantation for the salvage of infected total knee arthroplasty. J Bone Joint Surg Am. 1983;65(8):1087–98. 6630253

[4] Charette RS, Melnic CM. Two-stage revision arthroplasty for the treatment of prosthetic joint infection. Curr Rev Musculoskelet Med. 2018;11(3):332–40. 29948954 10.1007/s12178-018-9495-y PMC6105480

[5] Mian HM, Lyons JG, Perrin J, et al. A review of current practices in periprosthetic joint infection debridement and revision arthroplasty. Arthroplasty. 2022;4(1):31. 36045436 10.1186/s42836-022-00136-5 PMC9434893

[6] Hsieh PH, Chen LH, Chen CH, et al. Two-stage revision hip arthroplasty for infection with a custom-made, antibiotic-loaded cement prosthesis as an interim spacer. J Trauma. 2004;56(6):1247–52. 15211133 10.1097/01.ta.0000130757.53559.bf

[7] Kusuma SK, Ward J, Jacofsky M, Sporer SM, Della Valle CJ. What is the role of serological testing between stages of two-stage reconstruction of the infected prosthetic knee? Clin Orthop Relat Res. 2011;469(4):1002–8. 20941647 10.1007/s11999-010-1619-7 PMC3048278

[8] Lee YS, Fernando N, Koo KH, et al. What markers best guide the timing of reimplantation in two-stage exchange arthroplasty for PJI? Clin Orthop Relat Res. 2018;476(10):1972–83. 30794241 10.1097/01.blo.0000534680.87622.43 PMC6259852

[9] Aalirezaie A, Bauer TW, Fayaz H et al. Hip and knee section, diagnosis, reimplantation: proceedings of international consensus on orthopedic infections. J Arthroplasty. 2019;34(Suppl):S369–79. 10.1016/j.arth.2018.09.021 30343965

[10] Manthey N, Reinhard P, Moog F, et al. The use of [18F]fluorodeoxyglucose positron emission tomography to differentiate between synovitis, loosening and infection of hip and knee prostheses. Nucl Med Commun. 2002;23(7):645–53. 12089487 10.1097/00006231-200207000-00009

[11] Reinartz P, Mumme T, Hermanns B, et al. Radionuclide imaging of the painful hip arthroplasty. J Bone Joint Surg Br. 2005;87(4):465–70. 15795194 10.1302/0301-620X.87B4.14954

[12] Huang MJ, Hsieh PH, Ueng SW, et al. Use of positron emission tomography to detect infection around antibiotic-loaded cement Sp acers. Orthopedics. 2011;34(10):e605–9. 21956053 10.3928/01477447-20110826-08

[13] Gao Z, Jin Y, Chen X, et al. Diagnostic value of MRI lamellated hyperintense synovitis in periprosthetic infection of hip. Orthop Surg. 2020;12(6):1941–6. 33225607 10.1111/os.12789 PMC7767676

[14] Lee EM, Ibrahim EH, Dudek N, et al. Improving MR image quality in patients with metallic implants. Radiographics. 2021;41(4):E126–37. 34143712 10.1148/rg.2021200092

[15] Parvizi J, Zmistowski B, Berbari EF, et al. New definition for periprosthetic joint infection. Clin Orthop Relat Res. 2011;469(11):2992–4. 21938532 10.1007/s11999-011-2102-9 PMC3183178

[16] Chacko TK, Zhuang H, Stevenson K, Moussavian B, Alavi A. The importance of the location of FDG uptake in periprosthetic infection. Nucl Med Commun. 2002;23:851–5. 12195089 10.1097/00006231-200209000-00008

[17] Zhuang H, Duarte PS, Pourdehnad M, et al. The promising role of 18F-FDG PET in detecting infected lower limb prosthesis implants. J Nucl Med. 2001;42(1):44–8. 11197979

[18] Kwee RM, Kwee TC. 18F-FDG PET for diagnosing infections in prosthetic joints. PET Clin. 2020;15(2):197–205. 32145890 10.1016/j.cpet.2019.11.005

[19] Galley J, Sutter R, Stern C, et al. Diagnosis of periprosthetic hip joint infection using MRI. Radiology. 2020;296(1):98–108. 32396046 10.1148/radiol.2020191901

[20] Stambough JB, Curtin BM, Odum SM, et al. Does change in ESR and CRP guide timing of reimplantation? Clin Orthop Relat Res. 2019;477(2):364–71. 30566107 10.1097/01.blo.0000533618.31937.45 PMC6370077

[21] Chen SH, Ho KC, Hsieh PH, Lee MS, Yen TC. Potential role of FDG-PET/CT in detecting hip prosthesis infection. Q J Nucl Med Mol Imaging. 2010;54(4):429–35. 20823810

[22] Jones-Jackson L, Walker R, Purnell G, et al. Early detection of bone infection using FDG-PET. J Orthop Res. 2005;23(6):1484–9. 15896941 10.1016/j.orthres.2005.03.010.1100230635

[23] Koort JK, Makinen TJ, Knuuti J, Jalava J, Aro HT. Comparative FDG PET of osteomyelitis. J Nucl Med. 2004;45(8):1406–11. 15299068

[24] Desmouliere A, Redard M, Darby I, Gabbiani G. Apoptosis during transition between granulation tissue and Scar. Am J Pathol. 1995;146(1):56–66. 7856739 PMC1870783

[25] Gordon BA, Flanagan FL, Dehdashti F. Whole-body PET: pitfalls and considerations. AJR Am J Roentgenol. 1997;169(6):1675–80. 9393189 10.2214/ajr.169.6.9393189

[26] Purohit BS, Ailianou A, Dulguerov N, et al. FDG-PET/CT pitfalls in head and neck imaging. Insights Imaging. 2014;5(5):585–602. 25154759 10.1007/s13244-014-0349-x PMC4195840

[27] Subhas N, Patel PV, Pannu HK, et al. Imaging of pelvic malignancies with FDG PET-CT. Radiographics. 2005;25(4):1031–43. 16009822 10.1148/rg.254045155

[28] van der Bruggen W, Bleeker-Rovers CP, Boerman OC, et al. PET and SPECT in osteomyelitis and prosthetic infections. Semin Nucl Med. 2010;40(1):3–15. 19958846 10.1053/j.semnuclmed.2009.08.005

[29] Signore A, Sconfienza LM, Borens O, et al. Consensus document for diagnosis of prosthetic joint infections. Eur J Nucl Med Mol Imaging. 2019;46(4):971–88. 30683987 10.1007/s00259-019-4263-9 PMC6450843

[30] He C, Lu Y, Jiang M, et al. Optimized MRI for painful hip arthroplasty. Chin Med J (Engl). 2014;127(22):3876–80. 25421184

[31] Huang C, Chen Y, Ding H et al. Metal artifact reduction sequences MRI. J Clin Med. 2022;11(15). 10.3390/jcm11154371 PMC9369276 35955986

[32] Inaoka T, Kitamura N, Sugeta M, et al. Advanced metal artifact reduction MRI for PJI. J Comput Assist Tomogr. 2022;46(3):455–63. 35467584 10.1097/RCT.0000000000001297

[33] Henkelmann J, Henkelmann R, Denecke T, et al. Simultaneous FDG PET/MRI for PJI detection. Int Orthop. 2022;46(9):1921–8. 35635553 10.1007/s00264-022-05445-7 PMC9372014

[34] Arabi H, Zaidi H. Deep learning-based metal artefact reduction in PET/CT. Eur Radiol. 2021;31(8):6384–96. 33569626 10.1007/s00330-021-07709-z PMC8270868

[35] Sacher SE, Koff MF, Tan ET, et al. Advanced metal artifact reduction MRI in PJI. Skeletal Radiol. 2024;53(10):1969–78. 37875571 10.1007/s00256-023-04483-5 PMC11039568

[36] de Koster EJ, Vriens D, van Aken MO, et al. FDG-PET/CT cost-utility analysis. Eur J Nucl Med Mol Imaging. 2022;49(10):3452–69. 35435497 10.1007/s00259-022-05794-w PMC9308600

[37] Kurtz SM, Lau E, Watson H, Schmier JK, Parvizi J. Economic burden of PJI in the united States. J Arthroplasty. 2012;27(8 Suppl):61–e651. 22554729 10.1016/j.arth.2012.02.022

